# Photoprotective Properties of Isothiocyanate and Nitrile Glucosinolate Derivatives From Meadowfoam (*Limnanthes alba*) Against UVB Irradiation in Human Skin Equivalent

**DOI:** 10.3389/fphar.2018.00477

**Published:** 2018-05-15

**Authors:** Evan L. Carpenter, Mai N. Le, Cristobal L. Miranda, Ralph L. Reed, Jan F. Stevens, Arup K. Indra, Gitali Ganguli-Indra

**Affiliations:** ^1^Department of Pharmaceutical Sciences, College of Pharmacy, Oregon State University, Corvallis, OR, United States; ^2^Linus Pauling Institute, Oregon State University, Corvallis, OR, United States; ^3^Molecular and Cellular Biology Program, Oregon State University, Corvallis, OR, United States; ^4^Knight Cancer Institute, Oregon Health & Science University, Portland, OR, United States; ^5^Department of Dermatology, Oregon Health & Science University, Portland, OR, United States

**Keywords:** glucosinolate, isothiocyanate, nitrile, UVB, photoprotective, chemopreventive, 3D skin equivalent, MMPs (Min. 5–Max. 8)

## Abstract

Exposure to ultraviolet B (UVB) irradiation of the skin leads to numerous dermatological concerns including skin cancer and accelerated aging. Natural product glucosinolate derivatives, like sulforaphane, have been shown to exhibit chemopreventive and photoprotective properties. In this study, we examined meadowfoam (*Limnanthes alba*) glucosinolate derivatives, 3-methoxybenzyl isothiocyanate (MBITC) and 3-methoxyphenyl acetonitrile (MPACN), for their activity in protecting against the consequences of UV exposure. To that end, we have exposed human primary epidermal keratinocytes (HPEKs) and 3D human skin reconstructed *in vitro* (EpiDerm^TM^ FT-400) to UVB insult and investigated whether MBITC and MPACN treatment ameliorated the harmful effects of UVB damage. Activity was determined by the compounds’ efficacy in counteracting UVB-induced DNA damage, matrix-metalloproteinase (MMP) expression, and proliferation. We found that in monolayer cultures of HPEK, MBITC and MPACN did not protect against a UVB-induced loss in proliferation and MBITC itself inhibited cell proliferation. However, in human reconstructed skin-equivalents, MBITC and MPACN decrease epidermal cyclobutane pyrimidine dimers (CPDs) and significantly reduce total phosphorylated γH2A.X levels. Both MBITC and MPACN inhibit UVB-induced MMP-1 and MMP-3 expression indicating their role to prevent photoaging. Both compounds, and MPACN in particular, showed activity against UVB-induced proliferation as indicated by fewer epidermal PCNA+ cells and prevented UVB-induced hyperplasia as determined by a reduction in reconstructed skin epidermal thickness (ET). These data demonstrate that MBITC and MPACN exhibit promising anti-photocarcinogenic and anti-photoaging properties in the skin microenvironment and could be used for therapeutic interventions.

## Introduction

Exposure of the skin to ultraviolet (UV) radiation is a common aspect of human life; however, without appropriate caution, it can lead to undesirable skin aging and even cancer. These dermatological concerns arise due to a highly complex cascade of biochemical reactions that occur as stress responses in the skin attempt to counteract UV-induced damage. For that reason, it is necessary to develop better ways to not only block UV exposure but also ameliorate damage that does occur while also limiting detrimental physiological processes.

The clinical hallmarks of UV-induced photoaging include skin sagging, laxity, and wrinkling. At the molecular level, UV irradiation generates reactive oxygen species (ROS) which activate the transcription factor AP-1 responsible for regulating matrix-metalloproteinases (MMPs) and type I procollagen (Hanson and Clegg, 2002).

Increased collagen breakdown 24 h post-UV irradiation may be explained by the increased expression of MMP-1 (interstitial collagenase), MMP-3 (stromelysin-1), and MMP-9 (gelatinase-B; Quan et al., 2009). MMP-1 has been shown to mediate the proteolysis of dermal collagen fibrils and impairs the TGF-β pathway, altering the morphology of dermal fibroblasts (Xia et al., 2013). UV-induced activity of MMPs accounts for the loss of elasticity and flattening of the dermal–epidermal junction observed in photoaged skin.

The main mechanisms for photocarcinogenesis are DNA damage combined with elevated ROS. DNA damage occurs as UV rays are absorbed by DNA resulting in cyclobutane pyrimidine dimers (CPDs) and 6,4-photoproducts. Cancer results from mutations in key tumor suppressors such as p53 and/or in proto-oncogenes such as NRAS or BRAF in the case of melanoma (Einspahr et al., 1999; Hodis et al., 2012). In that process, ROS contribute by promulgating a chain of carcinogenic biochemical reactions (Bosch et al., 2015).

Phytotherapy, the use of plants for medicinal purposes, has been used in chemoprevention, mood disorder treatments, and dermatologic treatments to achieve promising outcomes. For instance, topical treatment of the polyphenolic compound (-)-epigallocatechin-3-gallate found in green tea confers protection against photocarcinogenesis by inhibiting ultraviolet B (UVB)-induced global DNA hypomethylation patterns (Xia et al., 2013). Saffron not only acts as a chemopreventive agent but also shows beneficial outcomes for patients with mild to moderate depression (Das et al., 2004; Jelodar et al., 2018). Pomegranate derived products have been shown to protect against UVB-induced CPDs and limit photoaging by modulating MMP expression and activity (Afaq et al., 2009). Therefore, natural products present a promising area for dermatological research as phyto-complexes are often hypoallergenic and can be absorbed by the superficial layers of the skin.

We present findings of a new source for naturally derived compounds that demonstrate photoprotective properties. The meadowfoam plant (*Limnanthes alba*) is native to the Pacific Northwest and is cultivated in the Willamette valley of Western Oregon as an oilseed crop. The seedmeal, a waste product of the oilseed industry, is a rich source of a single glucosinolate, glucolimnanthin (up to 4%). Enzymatic degradation of glucolimnanthin yields its derivatives, 3-methoxybenzyl isothiocyanate (MBITC) and 3-methoxyphenyl acetonitrile (MPACN), that have herbicidal properties (Stevens et al., 2009; Intanon et al., 2014). Using human primary epidermal keratinocytes (HPEKs) and three-dimensional human skin reconstructed *in vitro* (EpiDermFT), we sought to explore whether meadowfoam glucosinolate derivatives could ameliorate UVB-induced damage. Here we demonstrate for the first time that MBITC and MPACN exhibit photoprotective properties due to their activity in reducing UVB-induced DNA damage, proliferation, and MMP expression.

## Materials and Methods

### Chemicals

3-Methoxybenzyl isothiocyanate and MPACN were purchased from Oakwood Products, West Columbia, SC, United States, and from Sigma-Aldrich, St. Louis, MO, United States, respectively.

### Preparation of Augmented Meadowfoam Extract (ME) and Purified MBITC and MPACN From Meadowfoam Seed Meal

Meadowfoam seed meal was augmented by incubation with ground enzyme-active meadowfoam seed and water overnight at room temperature to convert the glucosinolate to isothiocyanate and nitrile metabolites. The augmented meal was freeze dried and then extracted with 95% HPLC-grade ethanol. The ethanolic extract was centrifuged to remove particulates and then assayed by HPLC with quantitation by UV absorption at 273 nm using commercial standards of MPACN and MBITC. The analysis confirmed that the glucosinolate was completely converted. For use in cell culture experiments, a concentrated stock solution of the extract was prepared by rotary evaporation to a target isothiocyanate level of near 50 mM. Analysis of the concentrated extract yielded values of 56.4 mM MBITC and 10.7 mM MPACN. A portion of the concentrated extract was fractionated using a 2 cm × 50 cm column of Sephadex LH-20 eluted with methanol to separate the MPACN and MBITC metabolites from other extract components.

### Cell Culture Studies

To test meadowfoam extract (ME) and ME-derived MBITC and MPACN for activity on mammalian cells, HACAT cells, seeded in 96-well plates, were treated with various concentrations (0.5–50 μM) of the test compounds. After a 24-h incubation with the test compounds, the media were aspirated and replaced with culture media containing MTT. Three hours later, the MTT media were aspirated and acidified isopropanol was added to the wells. The intensity of the blue formazan formed by cellular mitochondrial dehydrogenases was measured at 570 nm. For commercially available compound dose kinetics, HPEK cells (ATCC) were seeded 5000 cells/well into white, opaque 96-well plates (Greiner Bio-One) and allowed to incubate for 48 h. Afterward, cells were treated with 50–0.01 μM MPACN, MBITC, or ethanol vehicle control and incubated with the compounds for 12 h. Cells were then irradiated with 10 mJ/cm^2^ UVB using UVB TL 20W/12 RS M3 lights (Philips). UVB dose was quantified using an IL1400A NIST Traceable Photometer (International Light). After 36 h, CYQUANT (Thermo Fisher Scientific) assays were performed as per the manufacturer’s instructions. Cell counts were extrapolated from a standard curve of fluorescent signal activity vs. HPEK cell counts. Time kinetics involved the same compound and UVB treatment scheme as before, but CYQUANT assays were performed at 36, 60, or 84 h post-UVB irradiation. Cells were cultured in EpiLife medium (Thermo Fisher Scientific) supplemented with Human Keratinocyte Growth Supplement (Thermo Fisher Scientific). Incubations occurred in a humidified environment at 37°C with 5% CO_2_. Cells did not exceed 10 passages.

### Tissue UVB Irradiation and Sample Treatment

Following the protocol outlined by MatTek Corporation for tissue equilibration, EpiDerm^TM^ FT-400 was equilibrated in EFT media for 24 h at 37°C (5% CO_2_) prior to UVB irradiation. Samples were stored in six-well cultured plates, with the dermal side of the tissue in contact with media and the epidermal stratum corneum side exposed to the air. Two hours prior to UVB irradiation, topical application of 25 μL of MPACN and MBITC at 10, 25, and 50 μM was performed in triplicates. EFT culture media were replaced with PBS and samples were washed with PBS to remove any products that were not absorbed. Samples were then exposed to 60 mJ/cm^2^ of UVB radiation as was done with HPEK cultures. Immediately following irradiation, 25 μL of MPACN and MBITC was applied to respective samples, which were then cultured in fresh EFT media at 37°C for 48 h until harvesting.

### Histological Analyses

EpiDerm^TM^ FT-400 was gently isolated from their cassette, bisected, and fixed overnight in 4% paraformaldehyde in PBS. The samples were then dehydrated in graded series of ethanol and xylene before embedding in paraffin. Sections of 5 μm thickness were rehydrated and dissolved of paraffin before staining with hematoxylin and eosin. Images were taken with Leica DME microscope and Leica DFC280 digital camera. Epidermal thickness (ET) measurements were performed with Leica Application suite v3.30. Epidermal sunburned cells (SBCs) were counted per field using Image J software version 1.50i.

### Immunohistochemical (IHC) Analyses

Immunohistochemical (IHC) analyses were performed as previously described (Liang et al., 2012; Chagani et al., 2017; Li et al., 2017). Briefly, sections of 5 μm thickness were rehydrated and dissolved of paraffin with a graded xylene and ethanol series. Antigen retrieval was done with a citrate buffer pH 6.0. Blocking was done with 10% normal goat serum in PBS. Primary antibodies used were anti-CPD (Abcam, 1:1000) and anti-PCNA (Abcam, 1:6000) with incubation overnight at 4°C. Cy3 (Jackson Immuno Research, 1:400) was used as the secondary antibody. Nuclear staining was done using DAPI (0.2 ng/mL). Samples were then rinsed with PBST, dehydrated with graded ethanol and xylene washes, and mounted with distyene plasticizer xylene (DPX). Slides were allowed to dry overnight at room temperature before capturing images with Leica DMRA fluorescent microscope and Hamamatsu C4742-95 at 20x magnification using AxioVs40 version 4.8.2.0 software. Images were processed using Adobe Photoshop CC 2018 and Image J software version 1.50i.

### Immunoblotting and MMP Array

Immunoblotting was performed using denatured protein extracts taken from EpiDermFT cultures as previously described (Coleman et al., 2014; Guha et al., 2015). Total protein was quantified via BCA protein assay (Thermo Fisher Scientific). Membranes were probed for p-H2A.X (Cell Signaling Technology, 1:1000), MMP-1 (Santa Cruz Biotechnology, 1:200), MMP-3 (Santa Cruz Biotechnology, 1:200), MMP-9 (Santa Cruz Biotechnology, 1:100), Bax (Santa Cruz Biotechnology, 1:200), and cytoskeletal actin (Bethyl Laboratories, 1:2000) at 4°C overnight. Then membranes were probed with peroxidase conjugated Goat α-Rabbit (Calbiochem, 1:2000) or Goat α-Mouse (Calbiochem, 1:2000) and imaged with chemiluminescence reagents (GE Healthcare). Signal quantification was performed using ImageJ version 1.50i. The Human MMP Array C1 (RayBiotech) was loaded with 105 μg total protein from denatured extracts taken from skin equivalents treated under identical experimental conditions to those for western analyses and performed according to the manufacturer’s instructions.

### ELISA for MMP-9 Expression

Culture supernatants collected from HACAT cells simultaneously treated with TNFα and various concentrations of the test compounds were analyzed for MMP-9 expression using ELISA kits (eBioscience, San Diego, CA, United States) according to the manufacturer’s instruction manual.

### Zymography for MMP-9 and MMP-2 Activity

HACAT cells, seeded in 24-well plates, were treated with TNFα (10 ng/mL) and various concentrations (1, 5, and 10 μM) of ME. After a 24-h incubation, the cell culture supernatant was collected for gelatin zymography. The supernatant was mixed with Laemmli sample buffer with no reducing agents and loaded onto gels with gelatin for SDS-PAGE. After SDS-PAGE, the gels were stained with Coomassie Blue R-250, destained, photographed, and analyzed by ImageJ software.

### Statistical Analysis

Comparisons between the mean of two treatment groups were done using two-tailed Student’s *t*-tests. One-sample *t*-tests were performed to compare fold changes relative to vehicle control. All statistics were calculated using GraphPad Prism version 5.04.

## Results

### MBITC and MPACN Do Not Mitigate Effects of UVB on Cell Survival and Proliferation in a Monolayer Culture of Human Primary Epidermal Keratinocytes (HPEKs)

**FIGURE 1:**
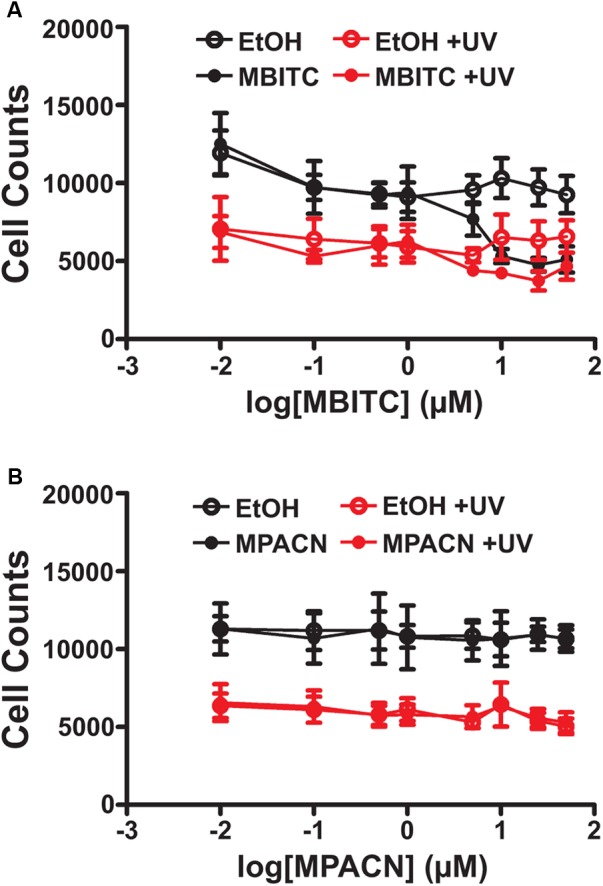
Effects of MBITC and MPACN on a human primary epidermal keratinocyte monolayer culture. HPEK cells treated with an increasing dose of either **(A)** MBITC, **(B)** MPACN, or ethanol vehicle control were then irradiated with a 10 mJ/cm^2^ dose of UVB. After 36 h, DNA content for each well was quantified using the CYQUANT assay and cell counts extrapolated from a standard curve of fluorescent signal intensity vs. HPEK cell count. Data represent mean ± SEM from three independent experiments performed in triplicate. MBITC caused a significant reduction in cell count at a 50 μM dose in the absence of UVB (*P* = 0.0471, two-tailed Student’s *t*-test).

We first sought to determine if augmented ME and MBITC and MPACN purified fractions of ME exhibited biological activity on mammalian cells due to its previously studied herbicidal properties. This was done by treating HaCaT cells with increasing concentrations of ME, MBITC, and MPACN. Addition of ME and MBITC fractions resulted in a significant reduction of HaCaT cell viability with increasing concentrations of the extracts (Supplementary Figure S1A). Subsequently, we focused on commercially available, purified MBITC and MPACN to determine activity in protecting HPEK cells against UVB insult in the absence of the skin microenvironment. To do this, we did a dose kinetics study by treating HPEK cells with an increasing concentration of either compound and then subjecting the cultures to a 10 mJ/cm^2^ dose of UVB irradiation which was based on our previous study (Hyter et al., 2013). Cell counts were then collected 36 h post-UVB treatment (Supplementary Figure S1B). We found that MBITC treatment did not ameliorate the reduced cell survival and proliferation observed after UVB irradiation, and furthermore it inhibited proliferation at a higher concentration in the absence of UVB (**Figure 1A**). MPACN also did not prevent UVB induced inhibition of cell proliferation; however, it did not have any effect on cell proliferation in the absence of UVB (**Figure 1B**). To determine if a longer time period was necessary to observe the effects of MBITC or MPACN treatment post-UVB irradiation, we performed a post-UVB time kinetics (Supplementary Figure S1C). We did not observe any additional effect of the compounds at 60 or 84 h (Supplementary Figures S1D,E).

### MBITC Reduces UVB Induced Hyperplasia in Human Reconstructed Skin *in Vitro*

**FIGURE 2:**
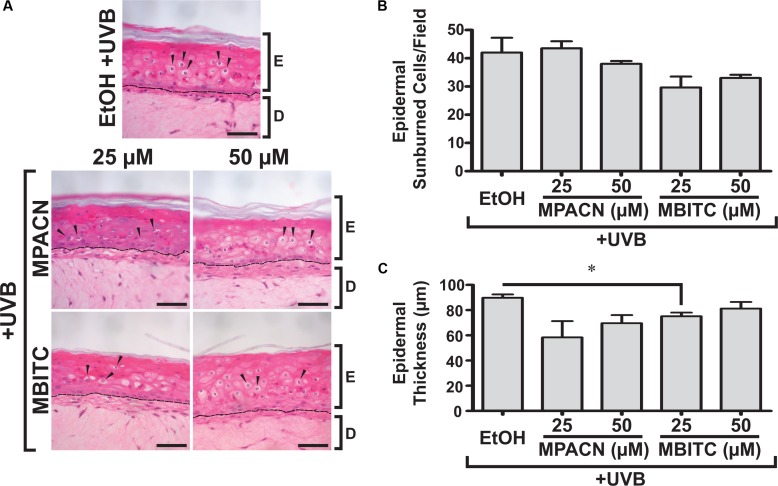
Hematoxylin and eosin staining of EpiDermFT human skin equivalent sections. EpiDermFT cultures treated with MBITC, MPACN, or ethanol vehicle control were then irradiated with a 60 mJ/cm^2^ dose of UVB. After 48 h, skin sections were collected and **(A)** H&E staining performed to quantify **(B)** epidermal sunburned cell counts and **(C)** epidermal thickness. Arrows indicate sunburned cells. Scale bars show 50 μm. Data represent mean ± SEM of three independent EpiDermFT cultures (*N* = 3). Significance is determined by two-tailed Student’s *t*-test. ^∗^*P* < 0.05.

We were then interested in testing MBITC and MPACN in a more biologically relevant model of *in vitro* reconstructed human skin. This model contains both epidermal and dermal portions and exhibits a similar response to UV as human skin. The presence of SBC after UVB-irradiation signals that the tissue has experienced UVB damage (Monteiro-Riviere et al., 2011). To examine whether MBITC or MPACN affords protection against this, compound-treated EpiDermFT cultures were exposed to a 60 mJ/cm^2^ dose of UVB and samples were collected after 48 h (Supplementary Figure S2). Histological analysis post H&E staining was performed to determine the presence of epidermal SBCs that have a characteristic morphology consisting of a pyknotic nucleus and eosinophilic cytoplasm (**Figure 2A**). Our results show a modest trend of decreased SBCs in samples treated with meadowfoam derived compounds (**Figure 2B**). In addition, UV radiation alters cell cycle machinery to lead to epidermal hyperplasia and subsequent tumorigenesis (Berton et al., 2001). The efficacy of compound treatment was further evaluated by examining ET post-UVB irradiation. Our results show decreased ET in samples treated with MPACN and MBITC with a significant reduction in ET observed for samples treated with 25 μM of MBITC (**Figure 2C**).

### MBITC and MPACN Decrease UVB-Induced DNA Damage Markers

**FIGURE 3:**
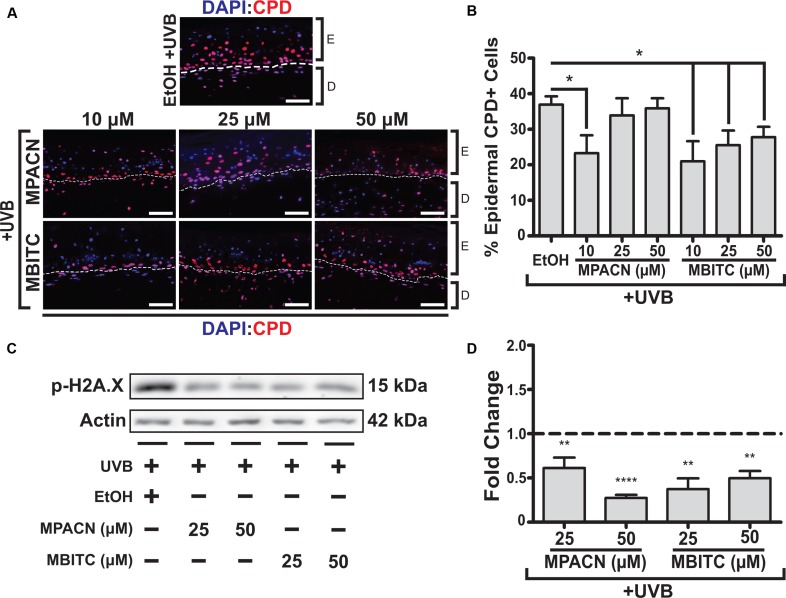
MBITC and MPACN cause a decrease in epidermal and total DNA damage markers. **(A,B)** Immunohistochemistry of MBITC, MPACN, and ethanol vehicle control treated EpiDermFT skin sections 48 h post-UVB irradiation to detect epidermal cyclobutane pyrimidine dimers. Data represent mean ± SEM for cell counts taken from three independent EpiDermFT cultures (*N* = 3). Scale bars indicate 50 μm. **(C,D)** Immunoblotting to probe for p-H2A.X in whole-culture denatured protein extracts. Shown is a representative blot and quantification – in fold change from ethanol control – for four independent experiments. Data represent mean ± *SD*. Significance is determined by two-tailed Student’s *t*-test **(B)** or by one-sample *t*-test for difference from one **(D)**. ^∗^*P* < 0.05, ^∗∗^*P* < 0.01, and ^∗∗∗∗^*P* < 0.0001.

CPDs arise due to absorption of UV by DNA, making them suitable markers for DNA damage caused directly by UVB irradiation. Therefore, we sought to examine the protective effects of MBITC and MPACN by examining the prevalence of CPDs in the epidermis. IHC was performed on paraffin sections using antibody against CPDs and CPD+ epidermal cell counts were collected (**Figure 3A**). A significant decrease in CPDs was found for all concentrations of MBITC tested and at a 10 μM dose for MPACN (**Figure 3B**). Interestingly, MPACN’s protection against CPDs did not appear to be dose-dependent as there appears to be an increasing trend in the mean value at a higher concentration. Total DNA damage was examined by looking at phosphorylated γH2A.X, which is a marker for DNA double-stranded breaks. A significant decrease in p-H2A.X was observed at all tested concentrations of MBITC and MPACN (**Figures 3C,D**). This suggests that both MBITC and MPACN show consistent protection against DNA damage either caused directly by UVB or by other mechanisms in response to irradiation.

### MBITC and MPACN Reduce UVB-Induced Proliferation

**FIGURE 4:**
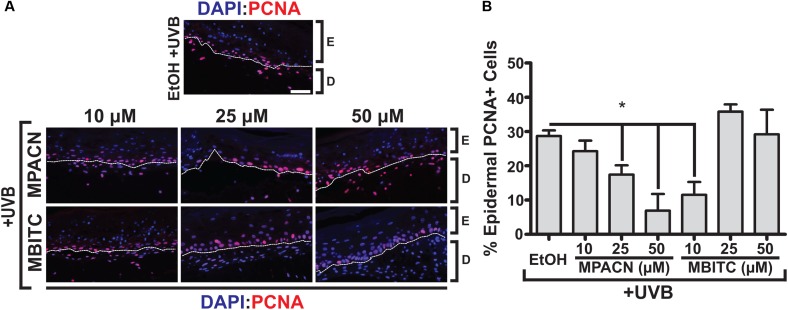
MBITC and MPACN decrease epidermal UVB-induced proliferation. **(A)** Immunohistochemistry to detect epidermal PCNA in sections from MBITC, MPACN, and ethanol vehicle control treated EpiDermFT cultures 48 h after a 60 mJ/cm^2^ dose of UVB irradiation. **(B)** Quantification of cell counts was taken from three independent EpiDermFT cultures (*N* = 3). Data represent mean ± SEM. Scale bars indicate 50 μm. Significance is determined by two-tailed Student’s *t*-test. ^∗^*P* < 0.05.

Elevated and sustained levels of proliferating cell nuclear antigen (PCNA) have been observed *in vivo* 48 h post-UVB irradiation in murine skin (Berton et al., 2001). PCNA is involved in DNA replication and repair with expression throughout the basal layer of skin (Moore et al., 2004). To investigate the effects of meadowfoam derived compounds in modulating UVB-induced proliferation in the epidermis, IHC using an antibody specific for PCNA was performed (**Figure 4A**). Our results indicate dose-dependent effects of MPACN on reduction of PCNA in UVB irradiated tissue (**Figure 4B**). An inverse relationship between decreasing PCNA signal and concentration of MPACN was observed, with concentrations of 25 and 50 μM of MPACN showing significant reduction in PCNA+ cells. The same trend was not observed for samples treated with MBITC. Rather, only a treatment of 10 μM of MBITC resulted in significant reduction of PCNA. As for cell death, we did not observe a significant change in expression of the pro-apoptotic marker Bax (Supplementary Figure S3).

### MBITC and MPACN Decrease UVB-Induced MMP-1 and MMP-3 Expression

**FIGURE 5:**
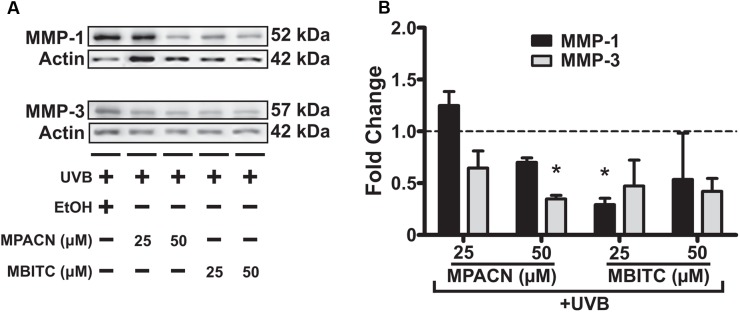
MBITC and MPACN cause a decrease in whole-culture matrix-metalloproteinases. **(A)** Immunoblotting for MMP-1 and MMP-3 in whole-culture denatured protein extracts from MBITC, MPACN, and ethanol vehicle control treated EpiDermFT cultures 48 h after a 60 mJ/cm^2^ dose of UVB. **(B)** Quantification of fold change relative to ethanol control from two independent experiments. Data represent mean ± *SD*. Significance is determined by a one-sample *t*-test for difference from one. ^∗^*P* < 0.05.

To get a global perspective of how MMP expression is affected by MBITC and MPACN after UVB treatment, we performed an MMP protein array on denatured protein extracts taken from compound and UVB treated EpiDermFT cultures (Supplementary Figure S4). UVB irradiation led to an increase in expression of number of MMPs and MPACN affected MMP-1 expression (Supplementary Figure S4C). To validate these data, we performed western blotting analysis (**Figure 5A**). We found that both compounds reduced expression of MMP1 and MMP3. A significant inhibition of MMP1 and MMP3 expression was observed after treatment with 25 μM of MBITC and 50 μM of MPACN, respectively (**Figure 5B**). Expression of MMP-9 was unaltered after treatment with MPACN or MBITC (Supplementary Figures S4D,E). The above results suggest that the meadowfoam glucosinolate derivatives inhibit expression of selective matrix metalloproteinases post-UVB irradiation.

## Discussion

Glucosinolate derivatives have been of interest to research for decades due to their chemopreventive properties as exemplified by sulforaphane. This isothiocyanate found in cruciferous (Brassicaceae) vegetables has been extensively studied, along with other isothiocyanate derivatives, for activity that includes modulating cyto-protective protein expression, inhibiting metastasis, epigenetic modification, activation of antioxidant mechanisms, and anti-inflammatory signaling (Kensler et al., 2005; Myzak et al., 2007; Hamsa et al., 2011; Kanematsu et al., 2011; Zhang et al., 2018). Sulforaphane has also been studied for its photoprotective properties though activation of the Nrf2 antioxidant pathway (Chaiprasongsuk et al., 2017). In the present study, we investigated whether derivatives of the meadowfoam glucosinolate, glucolimnanthin, also exhibit heretofore uninvestigated photoprotective properties. Our results demonstrate for the first time that MBITC and MPACN ameliorate UVB-induced DNA damage, modulate proliferation, and inhibit MMP expression in the skin microenvironment.

We have observed that MBITC and MPACN are unable to alleviate the impact of UVB irradiation on cell survival and proliferation of monolayer culture of HPEK cells. Another study has shown that in HaCaT and Hs27 cell lines, Nrf2 activators, including sulforaphane, were able to lessen the effects of UVB on cell survival (Tao et al., 2013). This suggests that MBITC and MPACN have either reduced potency for this activity or different mechanisms of action. We also found that MBITC inhibited proliferation of primary keratinocytes at a higher concentration, indicating possible cytotoxicity. Similar cytotoxic activity of sulforaphane has also been reported against HaCaT proliferation at concentrations above 5 μM (Shibata et al., 2010). It is interesting to note that the isothiocyanate glucosinolate derivatives demonstrate this activity while the nitrile derivative MPACN does not, suggesting distinct modes of action of the two derivatives.

In human reconstructed skin, MBITC and MPACN did not significantly reduce the number of epidermal SBCs. This is consistent with the HPEK data in that neither compounds appear to directly block UVB damage to cells to a significant degree. As before, Nrf2 activators were able to decrease the number of SBCs, indicating a consistent difference in mode of action of MBITC and MPACN from Nrf2 activators (Tao et al., 2013). However, MBITC could significantly and MPACN modestly reduce UVB-induced epidermal hyperplasia. This shows a similarity with sulforaphane’s anti-inflammatory properties which also cause reduced UVB-induced increases in ET *in vivo* (Shibata et al., 2010). We also observed that there did not appear to be a dose-dependent relationship between MBITC and ET as the effect was lost at a higher concentration. Our initial studies with HaCaT and HPEK cells have revealed dose-dependent toxic effects of MBITC at higher concentrations, although the same is not observed with MPACN. It is possible that similar adverse effects occur in cells within the 3D skin model and are observed at higher concentrations of MBITC. To replace the dead cells, keratinocytes need to proliferate and differentiate; this can lead, at least in part, to the modest increase in ET at a higher concentration of MBITC treatment. This observation is backed up by the lack of a dose-dependent decrease in epidermal PCNA+ cells with MBITC treatment.

We observed a consistent attenuation of UVB-induced DNA damage with our compounds as determined by reduced epidermal CPD+ cells and lower levels of total p-H2A.X in 3D skin equivalents. While our monolayer HPEK proliferation data indicated that these compounds do not prevent the direct effects of UVB insult, it does appear that MBITC and MPACN assist with the amelioration of incurred DNA damage 48 h post-UVB in a 3D model system. Interestingly, epidermal repair of CPDs appears to exist independent of the Nrf2 pathway. In mice that were either Nrf2 wildtype or null, the prevalence of CPDs post-UVB irradiation did not change (Kawachi et al., 2008). Indeed, even treatment with the Nrf2 activator bixin did not reduce CPDs in Nrf2 wildtype mice (Tao et al., 2015). These data further suggest differences between our compounds and Nrf2 activators. We also observed that the nitrile derivative MPACN was less effective in reducing epidermal CPDs at higher concentrations. This behavior bears a similarity to sulforaphane’s activity in reducing DNA damage incurred through oxidative stress. Sulforaphane appeared to promote DNA damage at higher concentrations perhaps due to its cytotoxicity on monolayer cultures of mesenchymal stem cells (Zanichelli et al., 2012). However, due to differences between MPACN and sulforaphane, this could be occurring through a separate mechanism. One potential explanation for this observation is that MPACN, whose concentration in the nucleus would be expected to increase with dose, functions to photosensitize adjacent pyrimidine nucleobases to undergo cyclobutane dimerization. Such a photosensitization mechanism might involve ROS-mediated formation of dioxetane intermediates, as has been postulated for the formation of CPDs in the dark after UVB irradiation of melanocytes followed by exothermic energy transfer of high-energy melanin products to adjacent thymine and cytosine bases (Baader et al., 2015). This explanation is in agreement with the less prominent increase in CPD formation at higher concentrations of MBITC because the concentration of MBITC would not increase in the nucleus as much as MPACN due to MBITC’s reactivity as an electrophile toward proteins in the cell culture medium and in the cytosolic compartment. As for DNA double-stranded breaks as determined by p-H2A.X levels, it appears that sulforaphane has been studied mostly for its cytotoxic properties of inducing apoptosis in cancer cell lines resulting in increased p-H2A.X levels (Singh et al., 2004). However, in large bowel organoids, sulforaphane was determined to have no effect on DNA double-stranded breaks due to oxidative stress (Frick et al., 2018). Altogether, our data indicate that MBITC and MPACN have a unique activity in reducing DNA damage when compared to sulforaphane.

Epidermal UVB-induced proliferation was inhibited by both MBITC and MPACN. However, the rate of reduction in PCNA+ cells was distinct between the two compounds. MBITC exhibited minimal effects on proliferation at higher concentrations while MPACN showed a dose-dependent decrease. Interestingly, the decreases in % epidermal proliferation observed for MPACN appeared inversely opposite to its effect in % CPD+ cells. A possible explanation for this is that at concentrations sufficient to reduce CPDs, epidermal cells more rapidly re-enter the cell cycle as they are have less UVB-induced DNA damage. Where CPDs are not attenuated, it is possible that epidermal cells require more time to repair DNA damage before engaging in mitosis. In the case of MBITC, we did not notice a similar relationship between CPDs and proliferation. At a 10 μM dose, MBITC reduced both DNA damage and proliferation while at higher concentrations, proliferation was not reduced compared to the control. It is possible that its potential cytotoxicity played a part to positively impact cell proliferation as previously mentioned. We did not observe any difference in expression of pro-apoptotic marker BAX in the MBITC treated samples compared to vehicle control, indicating no significant increase in cell death even at higher concentrations of MBITC treatment through this mechanism of action, although other changes in other cell-survival pathways cannot be ruled out. Altogether, our results suggest that at higher concentrations, MBITC exhibits cytotoxicity and might trigger cell death. To compensate for the loss of the cells due to toxicity, cells need to proliferate and differentiate, which is reflected in increased ET and increased epidermal PCNA+ cells. Thus, it is important to define an appropriate concentration range for both MPACN and MBITC treatment that maximizes the beneficial effects while eliminating the harmful and undesirable side effects.

As an indicator of photoprotection, we observed that MBITC and MPACN both inhibit MMP-1 and MMP-3 expression. Significant inhibition of MMP-1 expression by MBITC makes it a promising compound for preventing photoaging of the skin, whereas MPACN’s ability to significantly inhibit expression of MMP-3 makes it potentially beneficial in preventing invasion and metastasis of epidermal skin cancers, e.g., squamous cell carcinoma. The MMP inhibitory activity of isothiocyanates is also found in sulforaphane which has also been shown to inhibit MMP-1 (Chaiprasongsuk et al., 2017). In cancers, isothiocyanates have been found to downregulate MMP-9 expression and activity (Jeong et al., 2017). It is possible that in reconstructed skin, MMP-9 is not highly expressed post-UVB as seen in our MMP array data, which may mean that in a different context, MBITC and MPACN may modulate MMP-9 to a higher degree. Indeed, we found that in HaCaT cells stimulated with TNFα, that MMP-9 expression was significantly decreased with augmented ME and MBITC treatment. ME also reduced MMP-9 activity (Supplementary Figure S5). A further mechanistic understanding of the mode of action for MPACN and MBITC is necessary to contemplate the similarities and differences between the two glucosinolate derivatives in a cell and context specific manner. Altogether, both MPACN and MBITC hold promise to prevent UVB induced photo-aging and photo-carcinogenesis.

## Author Contributions

EC, AI, and GG-I designed the experiments. EC, ML, and GG-I performed the experiments. JS, AI, CM, RR, and GG-I provided technical and material support. EC, ML, JS, AI, and GG-I wrote the manuscript. All authors reviewed the manuscript.

## Conflict of Interest Statement

Natural Plant Products, Inc., Salem, Oregon, provided partial support for this study. The reviewer ZJ and handling Editor declared their shared affiliation.
