# Supplementary figures and images for: Photoprotective Properties of Isothiocyanate and Nitrile Glucosinolate Derivatives From Meadowfoam (Limnanthes alba) Against UVB Irradiation in Human Skin Equivalent
Source: Front Pharmacol. 2018 May 15;9:477. doi: 10.3389/fphar.2018.00477 (PMC5962701; doi:10.3389/fphar.2018.00477)

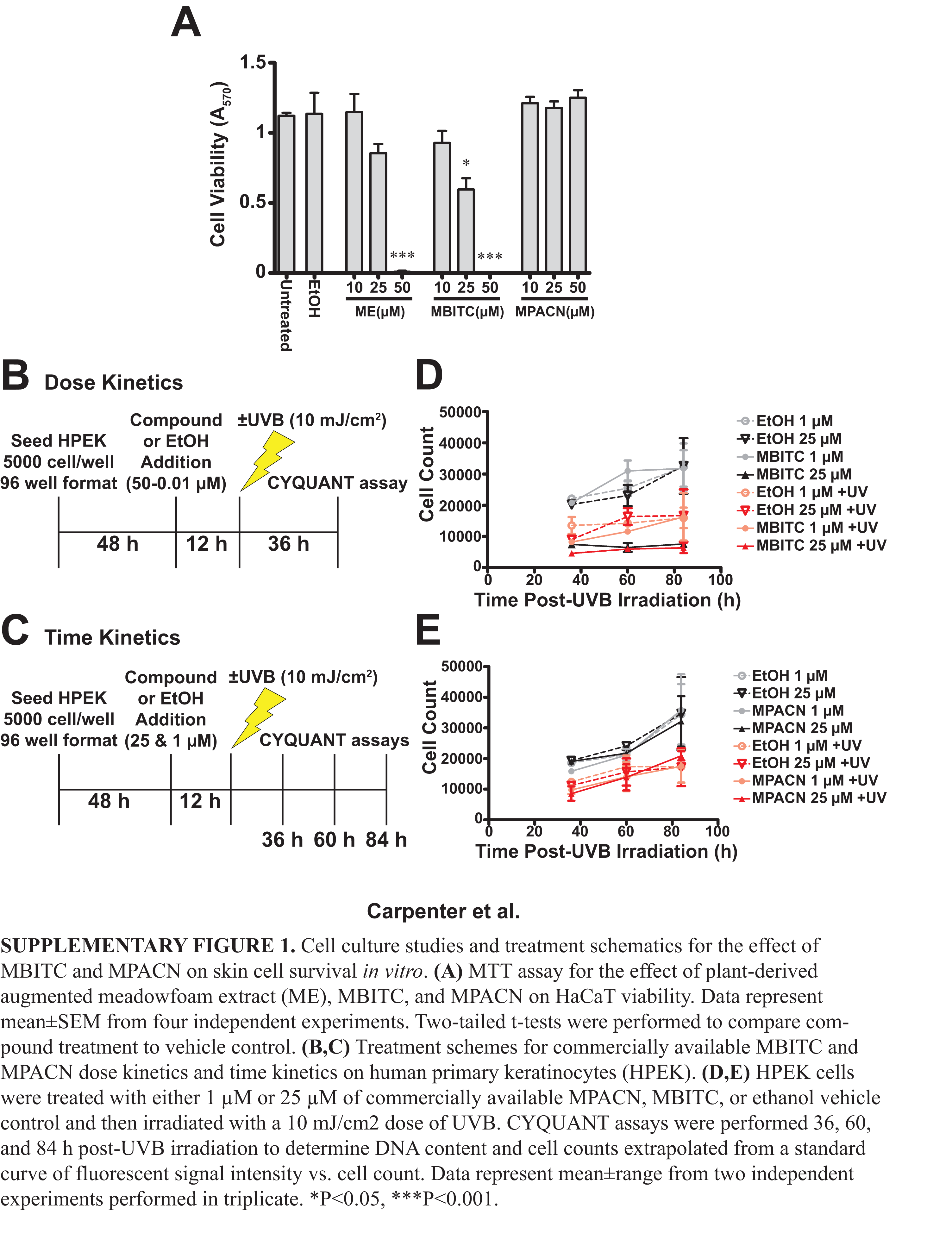

Supplement: Supplementary file 1 [file Image_1.TIF]

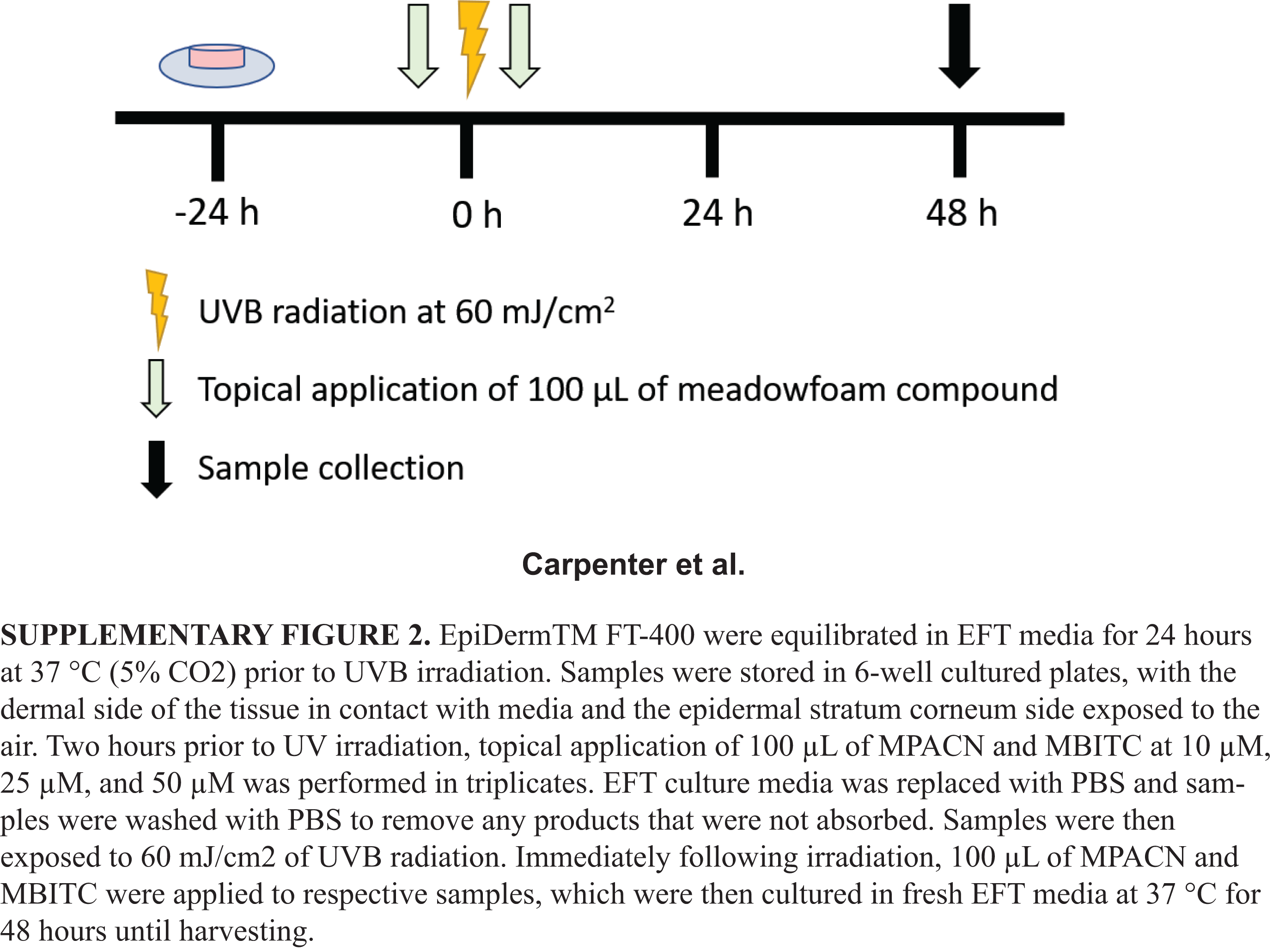

Supplement: Supplementary file 2 [file Image_2.TIF]

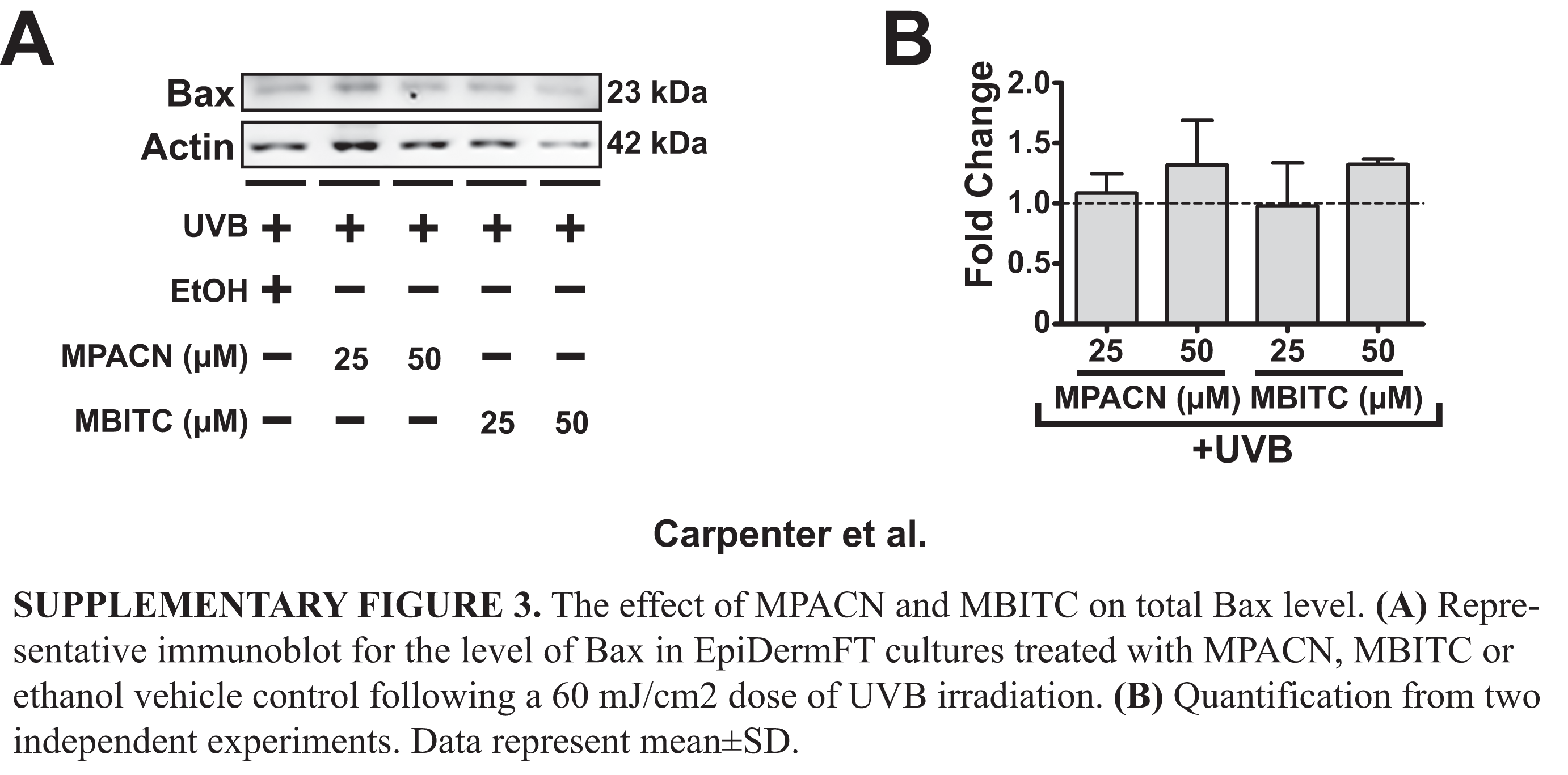

Supplement: Supplementary file 3 [file Image_3.TIF]

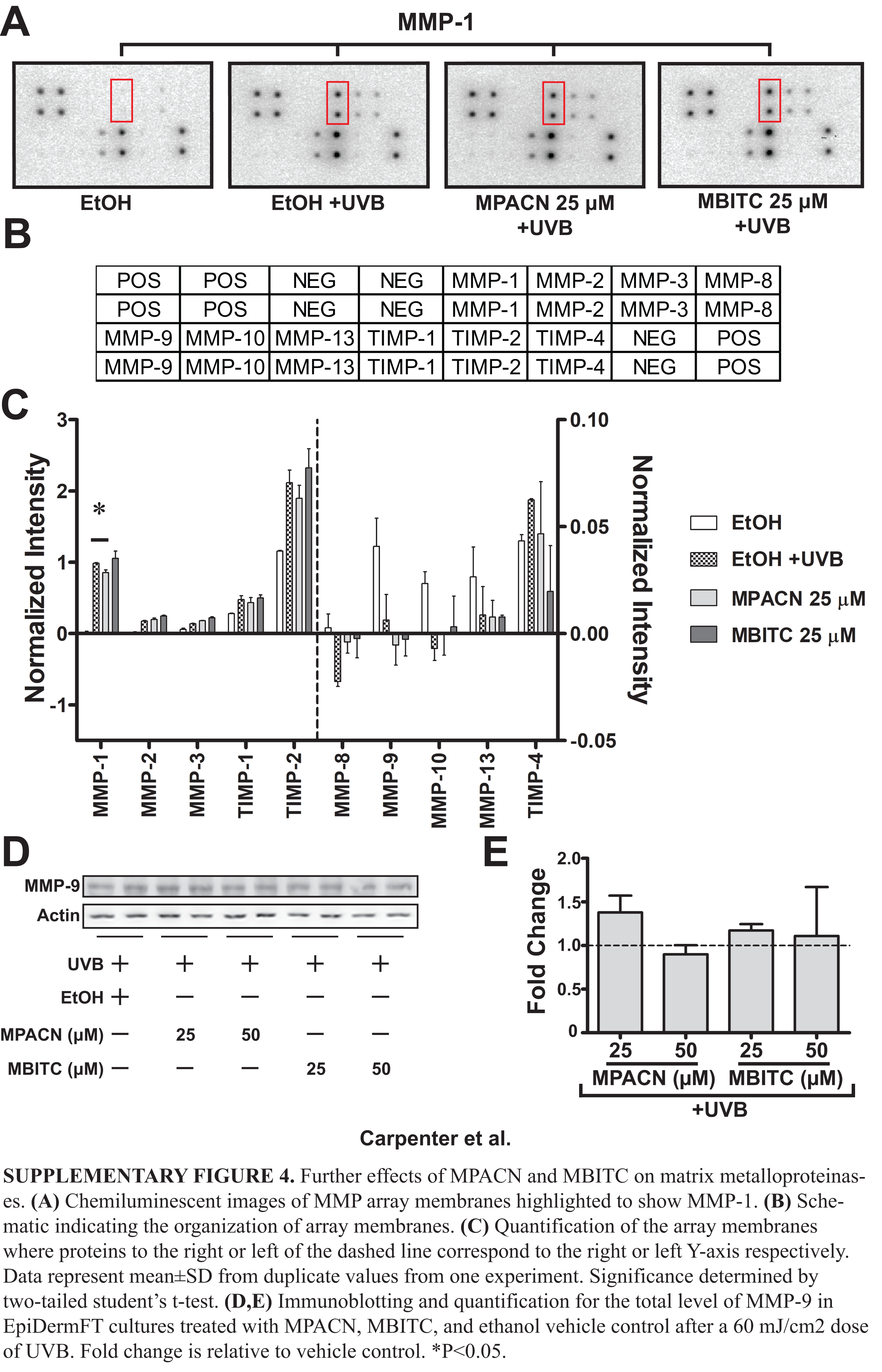

Supplement: Supplementary file 4 [file Image_4.TIF]

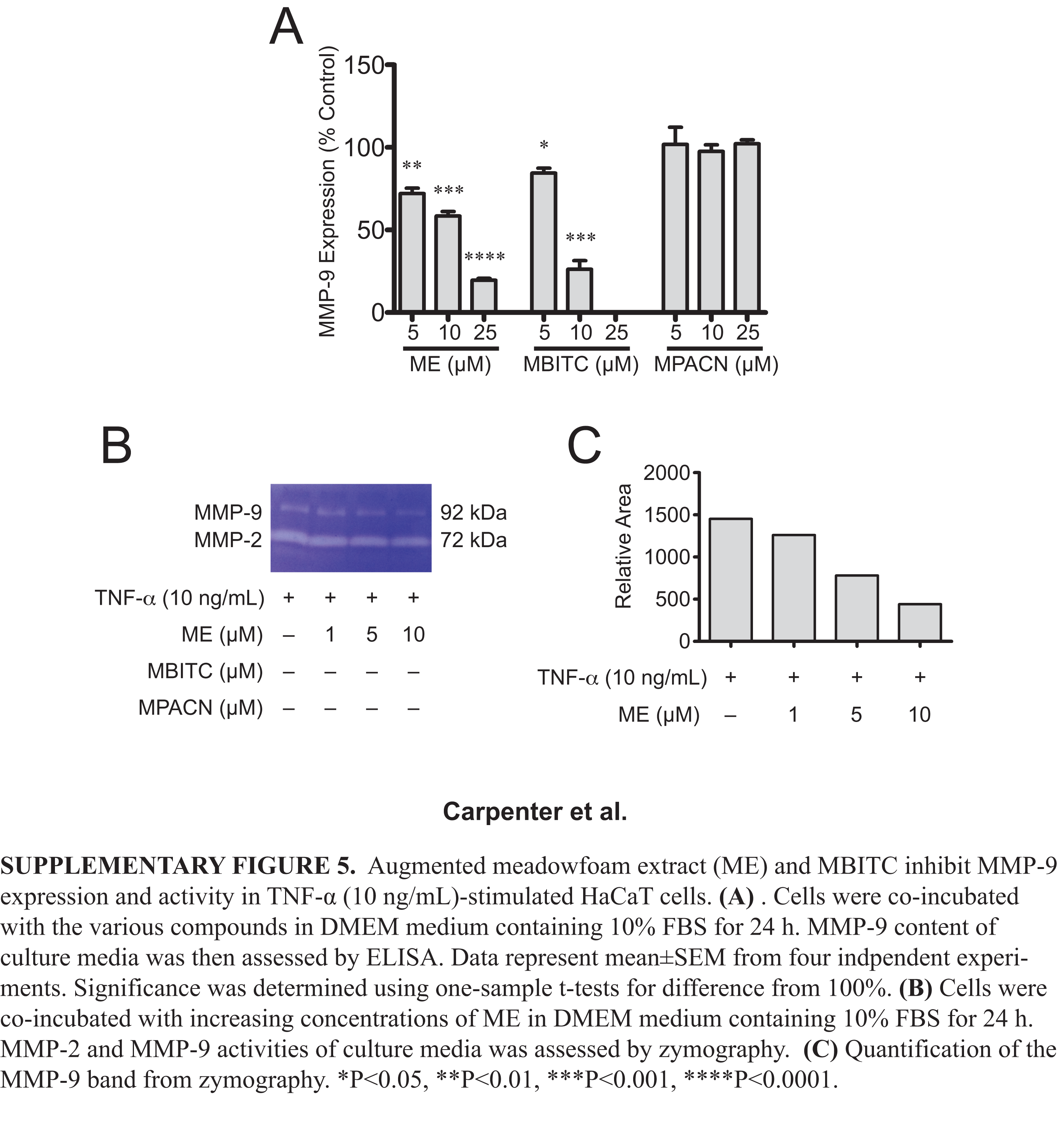

Supplement: Supplementary file 5 [file Image_5.TIF]
